# Different Biological Activities of Histidine-Rich Peptides Are Favored by Variations in Their Design

**DOI:** 10.3390/toxins13050363

**Published:** 2021-05-20

**Authors:** Morane Lointier, Candice Dussouillez, Elise Glattard, Antoine Kichler, Burkhard Bechinger

**Affiliations:** 1 Université de Strasbourg, CNRS, UMR7177, Institut de Chimie, 4, Rue Blaise Pascal, 67070 Strasbourg, France; lointier@unistra.fr (M.L.); glattard@unistra.fr (E.G.); 2 Laboratoire de Conception et Application de Molécules Bioactives, UMR7199 CNRS-Université de Strasbourg, Faculté de Pharmacie, 67401 Illkirch, France; c.dussouillez@unistra.fr; 3 Institut Universitaire de France, 75005 Paris, France

**Keywords:** saporin, luciferase, cell penetrating peptide, amphipathic helix, histidine, hydrophilic angle, protein delivery, antibacterial activity

## Abstract

The protein transduction and antimicrobial activities of histidine-rich designer peptides were investigated as a function of their sequence and compared to gene transfection, lentivirus transduction and calcein release activities. In membrane environments, the peptides adopt helical conformations where the positioning of the histidine side chains defines a hydrophilic angle when viewed as helical wheel. The transfection of DNA correlates with calcein release in biophysical experiments, being best for small hydrophilic angles supporting a model where lysis of the endosomal membrane is the limiting factor. In contrast, antimicrobial activities show an inverse correlation suggesting that other interactions and mechanisms dominate within the bacterial system. Furthermore, other derivatives control the lentiviral transduction enhancement or the transport of proteins into the cells. Here, we tested the transport into human cell lines of luciferase (63 kDa) and the ribosome-inactivating toxin saporin (30 kDa). Notably, depending on the protein, different peptide sequences are required for the best results, suggesting that the interactions are manifold and complex. As such, designed LAH4 peptides assure a large panel of biological and biophysical activities whereby the optimal result can be tuned by the physico-chemical properties of the sequences.

## 1. Introduction

Peptides are natural polymers that act as hormones, are used for signaling or are part of defense mechanisms against bacterial, fungal or viral infections [1,2,3]. As a consequence, there are many possible biomedical applications involving peptides or the mimetics thereof. Importantly, peptides and their building blocks are biodegradable, they can be produced in a reproducible and scalable manner, and procedures assuring analytical control of the products and their quality are well established. Chemical and biochemical reactions of amino acid side chains or termini that reproduce or mimic the naturally occurring posttranslational modifications of polypeptides, such as acetylation or phosphorylation, as well as the incorporation of non-natural amino acids further enhance the range of structures with highly variable characteristics. Furthermore, molecules have been created that represent the physico-chemical properties of known peptides [4,5,6,7]. These have been designed to be less prone to proteolytic degradation, thereby further expanding the range of possible applications.

Cecropins and magainins were among the first peptides from higher organisms for which a wide range of antimicrobial activities was described [8]. Early on after their discovery, biophysical approaches showed that these peptides are aligned along the surface of lipid bilayers rather than forming channels made of transmembrane helical bundles, as had initially been considered [9,10]. To further investigate the interaction contributions that govern peptide–membrane interactions, histidine-rich peptides were designed by assembling an alanine–leucine hydrophobic core, interspersed with four histidines (LAH4) that all align at one face in a helical conformation, and two lysines at each terminus [11]. As the pK_a_ values of these histidines are in the physiological range [12], changes in pH can be used to modulate the peptides’ amphipathicity and membrane topology. Indeed, under acidic conditions, the so-called LAH4 peptides align parallel to the phospholipid bilayer surface, but are transmembrane at neutral pH [11,13]. In their membrane surface orientation, they have been shown to associate into mesophase arrangements resulting in a high local density of peptides [14]. The interactions of the peptides with the bilayer interface results in a decreased order parameter of the phospholipid fatty acyl chains, considerable curvature strain on the membrane [15], pore-formation [16], and ultimately membrane lysis [17]. As expected from their initial design using cationic antimicrobial peptides as templates, members of the LAH4 family turned out to exhibit potent antimicrobial but also antiplasmodial activities, in particular at low pH, when they align parallel to the membrane surface [18,19,20]. 

In subsequent studies, additional biological activities were discovered for the LAH4 peptides. For example, they were found to be efficient vectors to transfer DNA or siRNA into eukaryotic cells [21,22,23]. The formation of peptide–DNA transfection complexes, including the size, charge and molecular details of how they form in a non-covalent manner, have been investigated in some detail using biophysical and biochemical approaches [21,24,25,26]. A model emerges wherein complexes form at neutral pH, which are taken up via endocytosis [21]. Upon endosomal acidification, a large fraction of the peptide is released [25] and becomes available to lyse the endosomal membranes [17]. Thus, the nucleic acids reach the cytoplasm in a self-promoted pathway where the peptide is involved in non-covalent complex formation, highly efficient release, and membrane passage of the nucleic acids. 

Furthermore, LAH4 peptides are able to assure the transfer of a large range of different cargo into cells, including various polypeptides, quantum dots or adeno-associated viruses [27,28,29,30,31]. Finally, variants of the first LAH4 peptides have been screened and developed to enhance ex vivo lentiviral transduction, an approach already used in clinical settings for gene therapeutic approaches [32,33]. 

While in the context of vaccine delivery LAH4 has helped in vectorizing CpG oligonucleotides in conjunction with peptides or proteins to be processed for presentation by MHC-I receptors [28], functional avidin-β-galactosidase, 530 kDa in size, has also been delivered in another set of experiments [29]. Of note, protein delivery has a therapeutic potential for the transport of proteins, such as the tumor suppressor p53 or the apoptosis-inducing cytochrome c into cells, which could be useful in the fight against cancer. Therefore, protein delivery technologies have been tested by fusion or conjugation to cationic molecules [34,35,36,37,38,39], including cell-penetrating peptides [40,41,42].

Therefore, in this paper the transduction of proteins was further elaborated by investigating the transport-dependence of luciferase and saporin by two series of LAH4 peptides with varying hydrophilic angles concomitant with differences in hydrophobic moment and amphipathicity (Table 1 and Figure S1). Saporin is a cytotoxic protein with potential applications in cancer therapy [43,44,45]. To further test how the physico-chemical properties of the peptides affect biological activities, their antimicrobial activities were evaluated at acidic and neutral conditions. Finally, the sequence dependence of different activities, such as calcein release, antimicrobial action, DNA transfection, and protein and lentiviral transduction, will be discussed and compared to each other.

## 2. Results

### 2.1. Design of the Peptides

Since the initial design of LAH4 and the discovery of its biophysical and biological activities, two other peptides, LAH4-A4 and LAH4-L1, have revealed great potential for their viral transduction and DNA transfection enhancement, respectively [11,21,29,32,46]. To better understand their mechanisms of action and their biophysical properties, several variants have been investigated. Among them, two series of peptides, all isomers of LAH4, were designed. These peptides are composed of the same 26 amino acids: two lysines at both termini and a central core of eight alanines, ten leucines and four histidines to form the amphipathic helix. These isomers differ only by the number (n) of leucines or alanines located between the histidines when represented as a Schiffer–Edmundson helical wheel, i.e., their “hydrophilic” angle (angle subtended by the positively charged histidine residues at pH 5; Table 1 and Figure S1). The peptides of these series were named “LAH4-Ln” and “LAH4-An”, where n refers to the number of leucines or alanines along the hydrophilic face, respectively (Figure S1). For example, LAH4-A4 presents four alanine residues between the histidine pairs. This peptide has been found to be highly efficient in promoting lentiviral transduction and is also named Vectofusin-1 [32,46]. Table 1 shows the different sequences with leucines (green) and alanine (magenta) involved in the hydrophilic angle separated by histidines (cyan). The amino acid modifications from the original LAH4 sequence result in hydrophilic angles ranging from 60° to 180° (Table 1 and Figure S1).

All these peptides have been previously tested for their capacity to deliver plasmid DNA into cells [47]. Figure S2 shows the transfection activities of the LAH4 peptides from both series. The highest activity is observed for LAH4-L1 and -A1 and tends to diminish when the number of leucines in the hydrophilic window increases (Figure S2). The same trend is observed for LAH4-A1 to -A4, while intriguingly high levels of activity are observed for -A5 and -A6. 

### 2.2. Protein Transduction Activity

**Figure 1:**
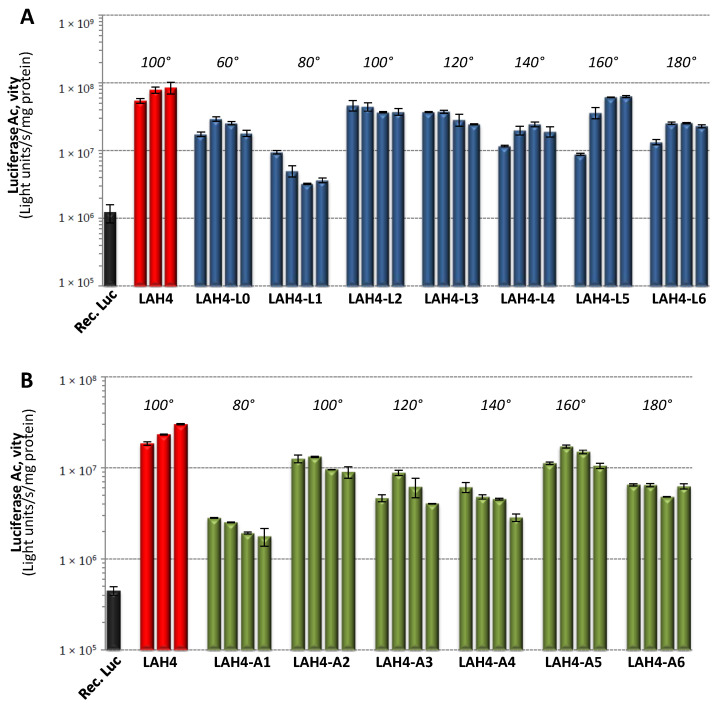
Transduction of the luciferase protein. HCT116 cells in 48-well plates were treated with 2 µg of recombinant luciferase (Rec. Luc) (per duplicate) in the presence or absence of LAH4 peptides (LAH4-Ln series in (**A**) and LAH4-An series in (**B**)). After 3 h of incubation in serum-free medium, cells were washed once with 1 mL/well of phosphate buffer saline (PBS), lysed, and the luciferase activity was determined. The transfection efficiency is expressed as light units/s/mg protein and the reported values are the mean of duplicates. Error bars represent the standard deviation of the mean. For each peptide, four conditions were tested: 10, 16, 24 and 32 µg of peptide/2 µg recombinant luciferase, except for LAH4 (16, 24 and 32 µg). Non-complexed recombinant luciferase was used as the control (black bar). The hydrophilic angles are shown in italics.

To perform a rapid screening of the protein transduction activities of the two series of peptides, we selected to deliver the luciferase protein from *Photinus pyralis*, which has a molecular weight (MW) of 62 kDa and an isoelectric point (pI) of 6.3. Indeed, the measurement of the activity of this enzyme is fast and very sensitive. However, it should be noted that this test does not allow us to distinguish the luciferase, which remains adsorbed on the cell surface from the one delivered inside the cells. Increasing amounts of peptides were added to a fixed amount of luciferase, and after 3 h of incubation with the human colon cancer cells HCT116, enzymatic activity was measured. The results show that of all the peptides tested, LAH4 was the most efficient (Figure 1). 

Interestingly, the LAH4-L1 and -A1 peptides that displayed the highest gene transfection capacities were among the least efficient for the delivery of the luciferase protein. The LAH4-An series and the LAH4-Ln series followed similar trends, although the LAH4-An showed lower transduction levels. Indeed, the A5/L5 and A2/L2 peptides were, together with LAH4, the most efficient peptides. 

Saporin from *Saponaria officinalis* is a 30 kDa protein that belongs to the family of ribosome-inactivating proteins (RIPs). RIPs have been widely studied because of their potential therapeutic application in a variety of human diseases as the toxic moiety of a conjugate. The conjugation of a cytotoxic RIP to a target-specific carrier, such as a monoclonal antibody (these conjugates are referred to as immunotoxins), allows the selective killing of target cells [43]. 

**Figure 2:**
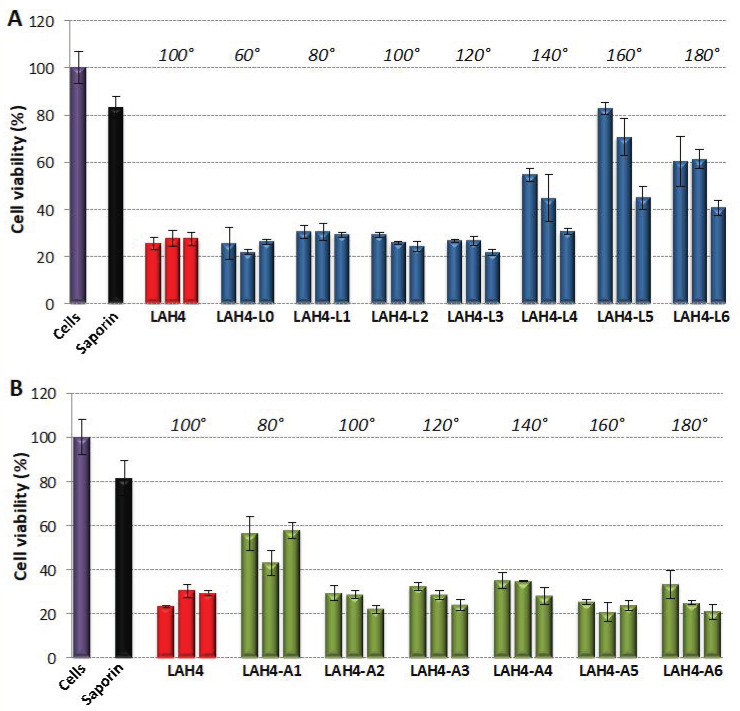
Transduction of the plant toxin saporin. HCT116 cells plated in 96-well plates were treated with 0.54 µg/triplicate of saporin in the absence or presence of LAH4 peptides (LAH4-Ln series in (**A**) and LAH4-An series in (**B**)). After 2 h 30 min of incubation the medium was replaced with a fresh one and the experiment was allowed to proceed. Two days later, an MTS assay was performed. The cell viability efficiency is expressed relative to untreated cells (=100%). Error bars represent the standard deviation of the mean. For each peptide 3 conditions were tested: 4.05, 5.4 and 8.1 µg of peptide/0.54 µg saporin. Untreated cells (=100%; purple bar) and non-complexed saporin (black bar) were used as the control. The hydrophilic angles are shown on top in italics.

Here, we asked whether LAH4 peptides could complex saporin in a non-covalent manner, deliver this protein into tumor cells and induce cell death. Of note, as saporin has an isoelectric point of 9.5, it has a global positive charge at physiological pH, meaning that the cationic peptides need to interact with the protein by other means than electrostatic interactions. The results show that saporin alone induces low levels of cell death, which is expected since it penetrates only poorly into cells. In contrast, when mixed with LAH4 peptides, cell viability could be reduced by up to 80% (Figure 2). 

For saporin transduction, the small hydrophilic angles of the L-series were the most active, whereas the activity profile shifted to the larger angles for the A-series. Overall, the large differences in activity between the An- and the Ln-series observed for luciferase transduction were less pronounced for saporin. To show that the cell killing activity came from saporin, the experiment was repeated with the LAH4 peptides alone, without saporin. The results shown in Figure S3 indicate that the peptides display no or only slight cytotoxicity on HCT116 cells.

Taken together, the results indicate that LAH4-derived peptides were able to deliver proteins of different molecular weight and different isoelectric points into cells. As a protein is unique by its structure and its sequence, it is not surprising that, in contrast to DNA, the optimal LAH4 variant has to be identified for each protein in order to obtain the highest delivery. 

### 2.3. Antibacterial Activity

**Figure 3:**
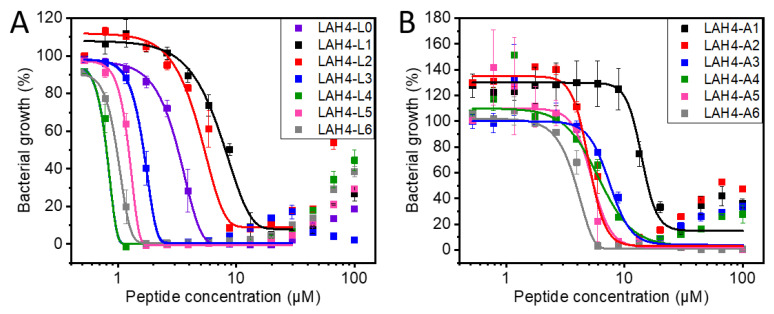
Bacterial growth as a function of peptide concentration for the LAH4-Ln (**A**) and the LAH4-An series (**B**). The average of two experimental data sets (each data point derived from triplicates under the same experiment conditions) covering the concentration range 100 µM to 0.52 µM are shown by the relative bacterial growth when compared to bacteria in the absence of peptide (=100%). To guide the eye, the data have been fitted with the function y_min_ + (y_max_ − y_min_)/(1 + (x/x_o_)^p^)^s^ using the OriginPro software. The decreased activity at the highest peptide concentrations is probably due to peptide aggregation [33], and these data were excluded from the fit. The assay was performed in Mueller–Hinton broth, pH 5.

The antimicrobial activity of LAH4 on the Gram-negative bacterium *E. coli.* was previously shown to be considerably more pronounced at pH 5 when compared to pH 7.5 [18]. Therefore, the antibacterial properties of both series of peptides were analyzed in Mueller–Hinton (MH) broth at pH 5 by determining the minimal inhibitory concentrations (MICs). Figure 3 shows the relative growth of *E. coli* cells as a function of peptide concentration. The peptide concentration where a minimum of bacterial propagation is reached (usually around 0–20%) was taken as the minimum inhibitory concentration (MIC) [48]. 

Figure 3A shows a wide distribution in the efficiency of the different peptides in inhibiting bacterial growth for the LAH4-Ln series. Indeed, while the most efficient peptide (LAH4-L4) has an MIC of 1 µM, the least efficient one exhibits an MIC of 20 µM (LAH4-L1). In comparison, most of the bacterial growth curves from the LAH4-An series exhibit MICs between 6 and 20 µM (Figure 3B). The MIC_50_ values derived from the measurements shown in Figure 3 are found in Table S1. 

**Figure 4:**
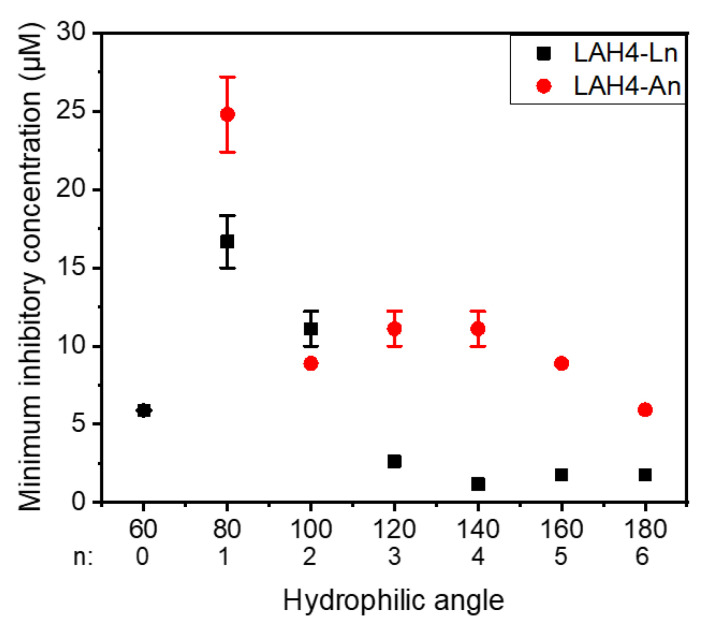
MIC as a function of the hydrophilic angle for the LAH4-An (red dots) and the LAH4-Ln series (black squares). For each peptide, the MIC is the concentration at which the bacterial growth reaches a minimum (usually around 0–20%). The assay was performed in Mueller–Hinton broth, pH 5.

Furthermore, the MICs were analyzed as a function of the hydrophilic angle (Figure 4). For both series, the highest MIC and thus lowest antimicrobial activity were observed for peptides covering a hydrophilic angle of 80° (25 µM for LAH4-A1 and 17 µM for LAH4-L1). Higher antimicrobial activity is observed when the angle increases. Indeed, for the LAH4-An series, little variation in the MIC is observed for hydrophilic angles between 100° and 180° (ranging from 5 to 12 µM). Of note, the MIC found for our reference peptide LAH4 (which has an angle of 100°) was 8.8 µM [18,49]. In contrast, for the LAH4-Ln series a continuous decrease in MICs is observed as the hydrophilic angle increases from 80° to 120°. For angles greater than 120° (LAH4-L4, LAH4-L5 and LAH4-L6) the MIC seems to reach a lower limit below 2 µM. The exception of this trend is LAH4-L0, whose MIC is as low as 6 µM despite a hydrophilic angle of only 60°. The best antimicrobial activities observed are the ones from the LAH4-Ln series with angles between 140° and 180°, where the MICs are ≤2 µM. 

## 3. Discussion

The LAH4 peptides have been designed to form amphipathic α-helical structures and to interact with membranes [11]. The peptides themselves are cationic, with a net positive charge of +5 at neutral pH and of +9 at acidic pH when the four histidines are protonated. Not unexpectedly, these cell-penetrating peptides preferentially interact with negatively charged lipids in membranes [22,50]. In lipid bilayers, LAH4 has been shown to adopt a transmembrane alignment at pH 7, whereas at acidic pH the highly charged LAH4 amphipathic helices orient along the surface and form higher-order mesophase arrangements [11,14]. Due to this variation in the membrane interaction mode, the peptides were investigated at different pH. Additionally, it has been shown that peptides of the LAH4 family exhibit antimicrobial effects [18,51,52], nucleic acid transfection [21,23], transduction of polypeptides and other cargo [29], and enhancement of lentiviral transduction activities [32,46,53]. In order to optimize the latter, two series of LAH4 sequences were designed and tested. When viewed as a helical wheel the peptides in a series differ in the angle subtended by the histidine residues with a defined number of either alanines or leucines defining the “hydrophilic face of the helix” (Figure S1) [32].

In this paper we investigate in considerable detail how some of the biological activities relate to the detailed sequence of the peptides. In particular, protein transduction, including luciferase and the toxin saporin, and the antimicrobial activities were studied in a systematic manner for two series of LAH4 sequences. The activities were analyzed as a function of the “hydrophilic angle” (Figure S1) and compared to previously studied lentiviral transduction [32], DNA transfection and calcein release efficiencies [47]. Interestingly, for many of the tested activities, the least efficient and optimal peptide sequences/hydrophilic angles can be identified (Figure S1 and Table 2 and Table 3). 

### 3.1. DNA Transfection 

The DNA transfection activity shows a maximum for peptides with hydrophilic angles around 80°, and this is also where the calcein release activities from POPE/POPG 3/1 (model for bacterial membranes) and POPC/POPS 3/1 (model membranes of eukaryotic cells) at pH 5 or pH 7.4 are highest [22,47]. Biophysical and biochemical investigations have allowed us to establish a model for the nucleic acid transfection activities. In a first step, peptide–nucleic acid complexes spontaneously form at neutral pH. Depending on the preparation method, these complexes exhibit diameters between 100 nm and several micrometers [21,24]. On average, about one peptide is electrostatically associated through the lysine side chains with two base pairs of DNA [25,26], resulting in a slightly positive surface charge of the complex [24]. Once the peptide/plasmid DNA transfection complexes are taken up, acidification of the endosomes results in additional positive charges for the peptides [21], a more relaxed packing of the complexes [54], and the release of almost half of the LAH4 peptides from DNA, which can thus interact with and destabilize the membranes [17,25]. Thereby, the model is in line—except for LAH4-A5—with the observed correlation between calcein release and transfection, where, in both cases, membrane pore formation and rupture are the limiting steps.

### 3.2. Antimicrobial Activities

**Figure 5:**
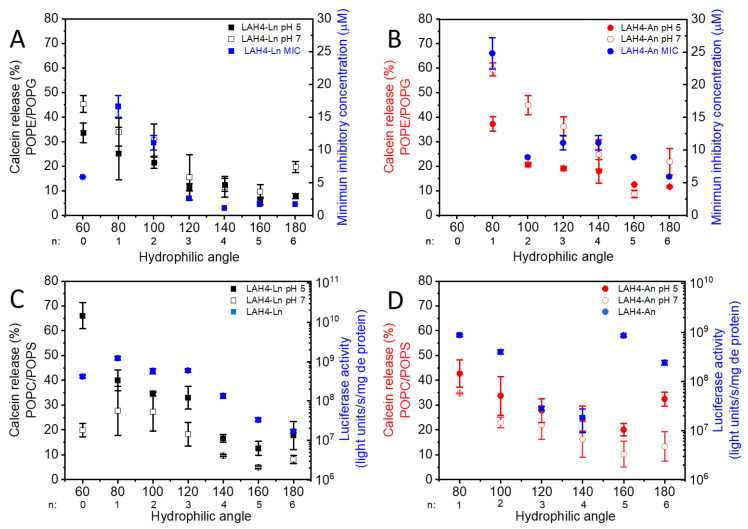
Calcein release as a function of hydrophilic angle and correlation with antimicrobial (**A**,**B**) or DNA transfection activities (**C**,**D**). Calcein release from LUVs composed of POPE/POPG (**A**,**B**) or POPC/POPS (**C**,**D**) is shown for the LAH4-Ln (black squares) and LAH4-An series (red circles) at pH 5 (filled symbol) and pH 7.4 (open symbol). Panels A and B also show the minimum inhibitory concentration (blue) for the LAH4-Ln series (**A**) and LAH4-An series (**B**). Panels C and D also show the luciferase activity after DNA transfection (blue) using the LAH4-Ln (**C**) or LAH4-An series (**D**).

The pronounced antimicrobial activities of cationic amphipathic peptides including LAH4 sequences have been associated with the perforation of microbial membranes [10,18,51,52,55]. Indeed, under conditions where the peptides align along the membrane surface [11,13,20], high concentrations of LAH4 peptides result in the lysis of lipid bilayers [17,22]. It is therefore unexpected that antimicrobial activity is highest for large hydrophilic angles, i.e., in inverse correlation with calcein release (Table 2, Figure 5). However, some of the naturally occurring antimicrobial peptides, such as cecropin and magainin 2, also have hydrophilic angles of 180° [56,57] (Figure S1). 

It has been shown that the efflux of small ions and the breakdown of the electrochemical gradient may be sufficient for bacterial cell killing [55]. This requires the formation of smaller/different openings, and thereby different physico-chemical interactions when compared to the release of larger molecules such as fluorescent dyes. Peptides with a large hydrophilic angle insert less deeply into the membrane, hydrophobic interactions become less pronounced, and membrane-association remains reversible. This observation is also in line with the better antimicrobial activity of the L-peptides (Figure 4), where the alanines accumulate on the hydrophobic face of the amphipathic helix (Figure S1). Furthermore, the leucines exposed to the hydrophilic surface potentially promote leucine zipper-like interactions between peptides inserted in opposing membranes, and thereby in agglutination of bacteria (and vesicles) [58,59]. Notably, a reduction in hydrophobic contributions has also been shown to result in less hemolytic side effects [60]. Other limiting factors for bacterial killing can be the passage across the bacterial outer membrane and/or the peptidoglycan layer [61]. Furthermore, the self-promoted passage of the peptides across the membrane [62] can result in antimicrobial activity developing inside the cell by, e.g., complexing polyanions [29,63,64].

### 3.3. Transfer of Proteins and Other Cargo

LAH4 peptides have not only been used to help nucleic acids penetrate into the cell interior, but also for the transfer of a variety of cargo, including proteins such as ovalbumin (44 kDa) and β-galactosidase of more than 500 kDa in size, pI < 5 [28,29]. This protein transduction activity was further investigated in this work (Figure 1 and Figure 2) by testing the transfer of luciferase from *Photinus pyralis* (62 kDa, pI 6.3) and of saporin from *Saponaria officinalis* (30 kDa, pI 9.5). Previously, it has been suggested that the transport and transfection properties of most cargo are based on the reversible formation of non-covalent complexes with the peptides at neutral pH, the pH-dependent release in the endosome and the membrane-lytic properties of LAH4 at acidic pH [25,29]. Note that the set of peptides that works best in promoting luciferase transduction (Figure 1, Table 2) is different from the one that helps saporin to develop high activity inside the cells (Figure 2). One may speculate that the surfaces of the proteins differ significantly, and concomitantly the interactions to form peptide–protein complexes require different physico-chemical and geometrical constraints. Thus, while electrostatic interactions dominate the reversible formation of complexes with nucleic acids [21,23,28] and remain highly important with membranes [29,50,59], other energetic contributions become important with proteins thereby favoring different sets of LAH4 peptides.

### 3.4. Lentiviral Transduction

Finally, lentiviral transduction is favored by yet other peptides, and follows other dependences on hydrophilic angle. Indeed, the LAH4-A4 peptide (with an angle of 140°) sticks out from the series because it enhances the transduction of VSV (vesicular stomatitis virus glycoprotein) and GALVTR (gibbon ape leukemia virus glycoprotein) pseudotyped lentiviruses, and has been protected under the name Vectofusin-1 [32,46]. Peptides of the LAH4-Ln series are in general less efficient in their transduction enhancement activities. As Vectofusin-1 shows the formation of fibers in the transfection and other phosphate-containing buffers [33], it has been speculated that this supramolecular assembly of the peptide is of critical importance to collect lentiviruses at the cellular surface, for the fusion of the viral and host cell membranes [46,65], and thus for transduction enhancement [33,66]. Thus, the hydrophilic angles and other peptide features that favor fiber formation may be key to this biological activity. Furthermore, similar considerations as for the recognition and transport of proteins may apply when it comes to identifying the peptides that can interact in the optimal manner with lentiviral receptors.

## 4. Conclusions

Peptides of the LAH4 family exhibit excellent antimicrobial, transfection, cell-penetrating and lentiviral transduction activities. These biological activities can be tuned through the manipulation of their sequence and hydrophilic angle. Whereas transfection and calcein release activities correlate well and agree with the idea that lysis of the endosomal membrane for release is the limiting factor, antimicrobial action shows an inverse relationship with the release of large fluorophores. The transport of other cargo or lentiviral transduction enhancement are promoted by yet other members of the LAH4 series. As such, each of these biological activities is characterized by different requirements in terms of the physico-chemical interactions of the peptides.

## 5. Materials and Methods

### 5.1. Materials

The LAH4 peptides (Table 1) were purchased from Pepceuticals (Leicestershire, UK). To exchange the counterions the peptides were solubilized three times in 2 mM hydrochloric acid and lyophilized. Some of the sequences were prepared in-house as described previously [29]. The purified peptides were stored at −20 °C.

### 5.2. Cell Culture

The human glioblastoma cell line U87 and the human colorectal carcinoma cells HCT116 were maintained in Roswell Park Memorial Institute (RPMI) 1640 medium containing 10% (*v*/*v*) FBS and penicillin–streptomycin (P/S; 10U/0.1 mg) in an incubator at 37 °C, 80% humidity and 5% CO_2_.

### 5.3. DNA Transfection Experiments

In vitro cell transfection experiments were performed using the plasmid p-Luc (7.6 kb), which is an expression plasmid encoding the *firefly luciferase* gene under the control of the human cytomegalovirus (CMV) immediate–early promoter. In total, 55,000 U87 cells were plated in 48-well plates one day before the experiment was performed. For transfection, 0.25 mL of serum-free culture medium containing the DNA complexes was deposited into each well of the duplicate. After incubation for approximately 2 h 30 min at 37 °C the medium was replaced with a fresh one containing serum. Luciferase activity was measured as described below in the cell lysate one day after transfection.

Transfection complexes were prepared as follows: for a duplicate, 1.5 μg of plasmid DNA and the desired amount of peptide were each diluted into 25 μL of acetate buffer pH 5 and gently mixed. DNA complexes were generated using increasing peptide/DNA *w*/*w* ratios. After 20 min of incubation at room temperature, the mixture was diluted with culture medium to obtain a final volume of 0.5 mL.

### 5.4. Luciferase Expression

For luciferase activity, cells were harvested in 100 μL of lysis buffer (8 mM MgCl_2_, 1 mM DTT, 1 mM EDTA, 0.6% Triton X-100, 15% glycerol, and 25 mM Tris-phosphate buffer pH 7.8). The cell lysate was then transferred into Eppendorf tubes and centrifuged for 7 min at 10,000× *g* to pellet debris. Luciferase light units were measured in a 96-well plate format with a Centro LB luminometer (Berthold) from an aliquot of the supernatant (2 μL) with 1 s integration after automatic injection of 50 μL assay buffer (lysis buffer without Triton X-100 but supplemented with 2 mM ATP) and 50 μL of a luciferin solution (167 μM in water). Luciferase background was subtracted from each value and the protein content of the transfected cells was measured by Bradford dye-binding using the BioRad protein assay (Bio-Rad). The transfection efficiency was expressed as light units/s/mg protein (light units measured over a period of 1 s, and the values were then normalized after measurement of the amount of protein present in each well). The reported values are the mean of duplicates. Error bars represent the standard deviation of the mean.

### 5.5. Luciferase Transduction Experiments

Preparation of the peptide/luciferase mixture: for a duplicate, 2 μg of recombinant luciferase (luciferase from *Photinus pyralis*, Sigma) and the desired amount of peptide were each diluted into 20 μL of PBS and gently mixed. Protein complexes were generated using increasing peptide/protein *w*/*w* ratios. After 30 min of incubation at room temperature, the mixture was diluted with serum-free culture medium to obtain a final volume of 0.5 mL.

Protein transduction: in total, 140,000 HCT116 cells were plated in 48-well plates one day before the experiment. Then, 0.25 mL of serum-free medium containing the peptide/protein complexes was transferred into each well. After an incubation time of 3 h at 37 °C the medium was removed, cells were washed once with 1 mL/well of PBS, and then 100 μL of lysis buffer was added per well. Luciferase activity and protein content were measured as described above.

### 5.6. Saporin Delivery

Formation of the peptide/saporin mixture: for a triplicate, 0.54 μg of saporin (from *Saponaria officinalis* seeds, Sigma) at 0.1 mg/mL in water was added to the desired amount of peptide diluted in 30 μL of PBS and the mixture was then gently mixed. Protein complexes were generated using increasing peptide/protein *w*/*w* ratios. After 30 min of incubation at room temperature, the mixture was diluted with serum-free culture medium to obtain a final volume of 0.225 mL.

Protein transduction: in total, 12,000 HCT116 cells were plated in 96-well plates one day before the experiment. Then, 75 µL of serum-free medium containing the peptide/protein complexes was transferred into each well of the triplicate. After an incubation time of 2 h 30 min at 37 °C the medium was removed and replaced with a fresh one containing serum. After 48 h of incubation, 20 µL/well of the MTS reagent was added. The cytotoxicity assay was then performed as described below.

### 5.7. Cytotoxicity Assay

Cell metabolic activity was measured by using an MTS (3-(4,5-dimethylthiazol-2-yl)-5-(3-carboxymethoxyphenyl)-2-(4-sulfophenyl)-2H-tetrazolium) assay (CellTiter 96^®^ AQ ueous One Solution Cell Proliferation Assay, Promega). MTS is a colorimetric reagent that undergoes color change in the presence of NAD(P)H, which is proportional to metabolic activity. Following treatment for the desired length of time, the MTS reagent was added according to the manufacturer’s instructions, followed by incubation at 37 °C for 30–60 min (until significant color change was observed). Plates were read at 490 nm (reagent absorbance) and 680 nm (background) (SP200, Safas, Monaco). The results were expressed by the subtraction of background absorbance and the expression of viability relative to non-treated controls (considered as 100% viability).

### 5.8. Antibacterial Activity

A broth microdilution assay was used to determine the minimal inhibitory concentration (MIC). A peptide aliquot of 1 mg was first solubilized in 50% ethanol (10 mM) then diluted in H_2_O to get a 300 μM peptide stock solution.

For all the activity tests, *E. coli* bacteria (ATCC^®^ 25922^TM^, Thermo Fisher Scientific, Courtaboeuf, France) were used. Briefly, some colonies from *E. coli* spread on gelose were resuspended in Mueller–Hinton broth, pH 7.4. After incubation at 37 °C for about 6 h this preculture was diluted to OD_550nm_ = 0.2 in MH at pH5 and allowed to grow to reach the exponential phase (OD_550nm_ = 1–2). After two-step dilution in MH pH5, the working bacteria suspension corresponding to 1.10^5^–2.10^5^ colony-forming units (CFU)/mL was obtained.

The antimicrobial testing was carried out in 96-well microplates (sterile polystyrene untreated with F-bottom, Thermo Scientific Nunc A/S, Roskilde, Denmark). The first column of the plate contains the highest final concentration (C_f_) of peptide to test (two experiments were done: the first one starting at C_f_ 100 μM and a second one starting at 30 μM). All samples were added to the first column of the plate and subsequently spread onto the plate by a 1.5-fold dilution series in 10 steps. The last columns correspond to the positive and negative control conditions, respectively. Finally, the bacterial working suspension was distributed (50 μL at 1.10^5^–2.10^5^ CFU/mL in MH1X at pH 5) to each well except for the negative control (MH1X at pH5). The final volume was 100 μL/well and the range of peptide concentrations tested was from 100 μM to 1.73 μM for the first experiment and from 30 μM to 0.52 μM for the second experiment. The microplates were incubated at 37 °C for 12 h before bacterial growth was assessed by measuring the OD_600_. The initial bacterial density for each experiment was checked by spreading 100 μL of the working bacterial suspension, diluted 1/100 and 1/500 in MH1X, pH 5 on MH agar plates. After overnight incubation at 37 °C, the CFU were counted and the CFU/mL were determined. The percentage of bacterial growth compared to the positive control allows one to determine the MIC. The MIC values represent the average of the lowest tested peptide concentration (from both experiments) where most (at least 80%) of the bacterial growth is inhibited.

## Figures and Tables

**Table 1:** Peptide sequences studied in this work and the corresponding hydrophilic angle when presented in a Schiffer–Edmundson helical wheel projection (see Figure S1). The histidine residues (in cyan) delineate the hydrophilic angle; the amino acids present between the histidines within the hydrophilic face are either leucines (green) or alanines (magenta).

Name	Sequences	Hydrophilic Angle
LAH4	NH_2_-KKALLALALHHLAHLALHLALALKKA-Amide	100°
LAH4-L0	NH_2_-KKALLAHALAHLALLALHLALHLKKA-Amide	60°
LAH4-L1	NH_2_-KKALLAHALHLLALLALHLAHALKKA-Amide	80°
LAH4-L2	NH_2_-KKALLALALHHLALLALHLAHALKKA-Amide	100°
LAH4-L3	NH_2_-KKALLALALHHLALLAHHLALALKKA-Amide	120°
LAH4-L4	NH_2_-KKALLHLALLHAALLAHHLALALKKA-Amide	140°
LAH4-L5	NH_2_-KKALLHLALLHAALLAHLAALHLKKA-Amide	160°
LAH4-L6	NH_2_-KKALLHLALLLAALHAHLAALHLKKA-Amide	180°
LAH4-A1	NH_2_-KKALLAHALHLLAALALHLAHLLKKA-Amide	80°
LAH4-A2	NH_2_-KKALLLAALHHLAALALHLAHLLKKA-Amide	100°
LAH4-A3	NH_2_-KKALLLAALHHLLALAHHLAALLKKA-Amide	120°
LAH4-A4	NH_2_-KKALLHAALAHLLALAHHLLALLKKA-Amide	140°
LAH4-A5	NH_2_-KKALLHALLAHLAALLHALLAHLKKA-Amide	160°
LAH4-A6	NH_2_-KKALLHALLAALLAHLHALLAHLKKA-Amide	180°

**Table 2:** The biological activities as a function of the hydrophilic angle. For each of the two series the most (+) and the least active (−) of the peptides are indicated in terms of their hydrophilic angle.

Peptide	Calcein Release (pH 5) ^1^		Antimicrobial Activity (pH 5)		DNA Transfection		Virus Delivery ^2^	
	+	−	+	−	+	−	+	−
LAH4-An	80°	160°	180°	80°	80°	140°	140°	100°/180°
LAH4-Ln	60°	160°	140°	80°	80°	180°	120°/140°	80°/180°

**^1^** Data from reference [47]. The best/worst angles were the same for the assays done with POPE/POPG 3/1 mole/mole or POPC/POPS 3/1 mole/mole liposomes. ^2^ Data from reference [32].

**Table 3:** The protein transduction activities as a function of the hydrophilic angle. For each of the two series the most (+) and the least active (−) of the peptides are indicated in terms of their hydrophilic angle.

Peptide	Luciferase Transduction		Saporin Delivery	
	+	−	+	−
LAH4-An	100°/160° *	80°	100–180°	80°
LAH4-Ln	100°/160° *	80°	60–120°	160–180°

* The best activities were obtained with the parent peptide LAH4.

## Data Availability

Data will be made available upon request.

## Supplementary Materials

The following are available online at https://www.mdpi.com/article/10.3390/toxins13050363/s1, Figures S1–S3: Helical wheel representations of the peptides, transfection efficiency of U87 cells and peptide toxicity. Table S1: MIC50 values.
