# Supplementary material for: Different Biological Activities of Histidine-Rich Peptides Are Favored by Variations in Their Design
Source: Toxins (Basel). 2021 May 20;13(5):363. doi: 10.3390/toxins13050363 (PMC8160934; doi:10.3390/toxins13050363)
Supplement: Supplementary file 1 [file toxins-13-00363-s001.zip › toxins-1223699 supp update.pdf]

Supplementary Materials: Different Biological Activities of Histidine-Rich Peptides Are Favored by Variations in Their Design

Morane Lointier, Candice Dussouillez, Elise Glattard, Antoine Kichler and Burkhard Bechinger

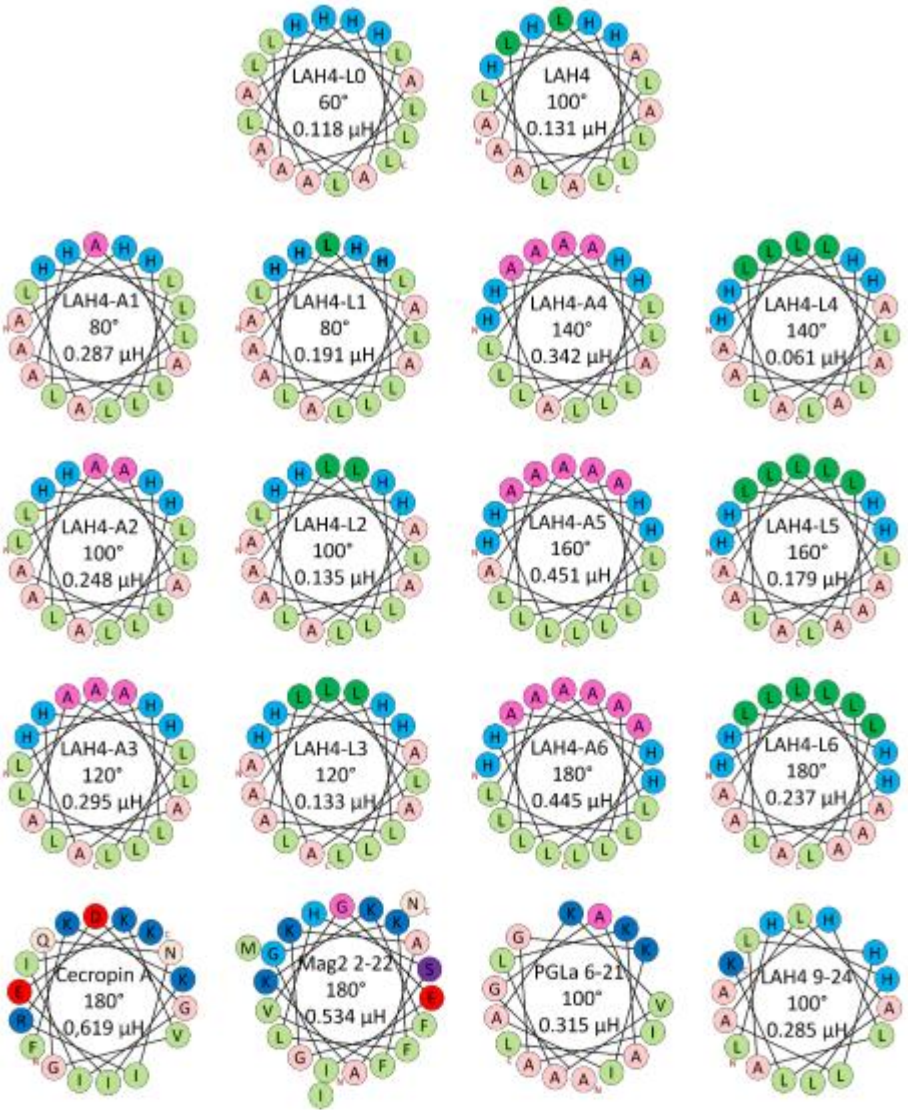

Figure S1. Schiffer-Edmundson helical wheel representations of the putative amphipathic α-helical region (residues 6–23) of the LAH4-An and LAH4-Ln peptides and their corresponding hydrophilic angle. The structured regions forming amphipathic helices of cecropin A, magainin 2, PGLa and LAH4 are also shown.

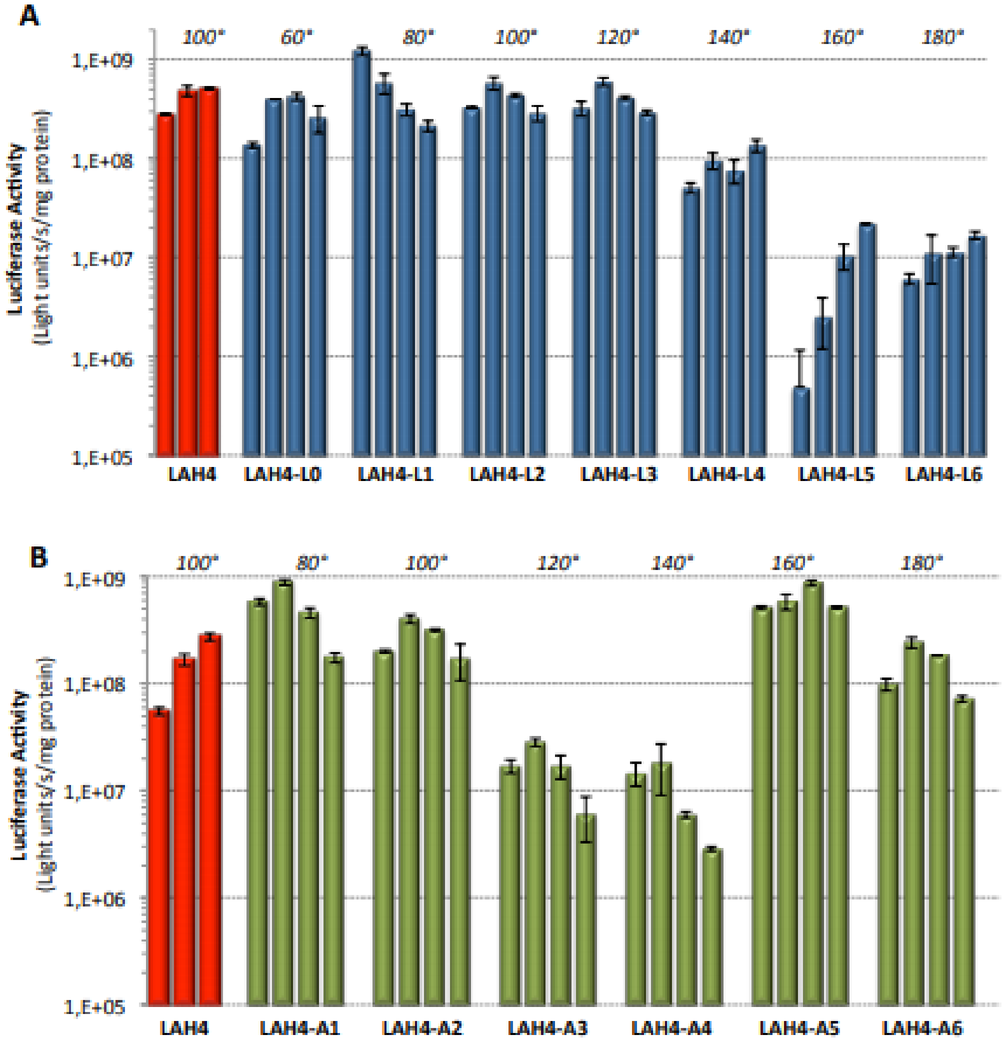

Figure S2. Transfection efficiency of the LAH4-An and LAH4-Ln peptides evaluated on human glioblastoma U87 cells. Increasing amounts of peptide were mixed with a constant amount of reporter plasmid (1.5 µg per duplicate of pDNA) and the complexes were incubated for 2h 30min with the cells plated in 48-well plates. The transfection medium was then removed and replaced with fresh culture medium supplemented with 10% serum. Luciferase activity was measured one day post-transfection. The transfection efficiency is expressed as light units/s/mg protein and the reported values are the mean of duplicates. Error bars represent the standard deviation of the mean. In italics are shown the hydrophilic angles. For each peptide, except LAH4 (10, 18 and 24 µg for graph **A** and 7.5, 10 and 18 µg for graph **B**), four conditions were tested: 7.5, 12, 18 and 25 µg of peptide/1.5 µg DNA.

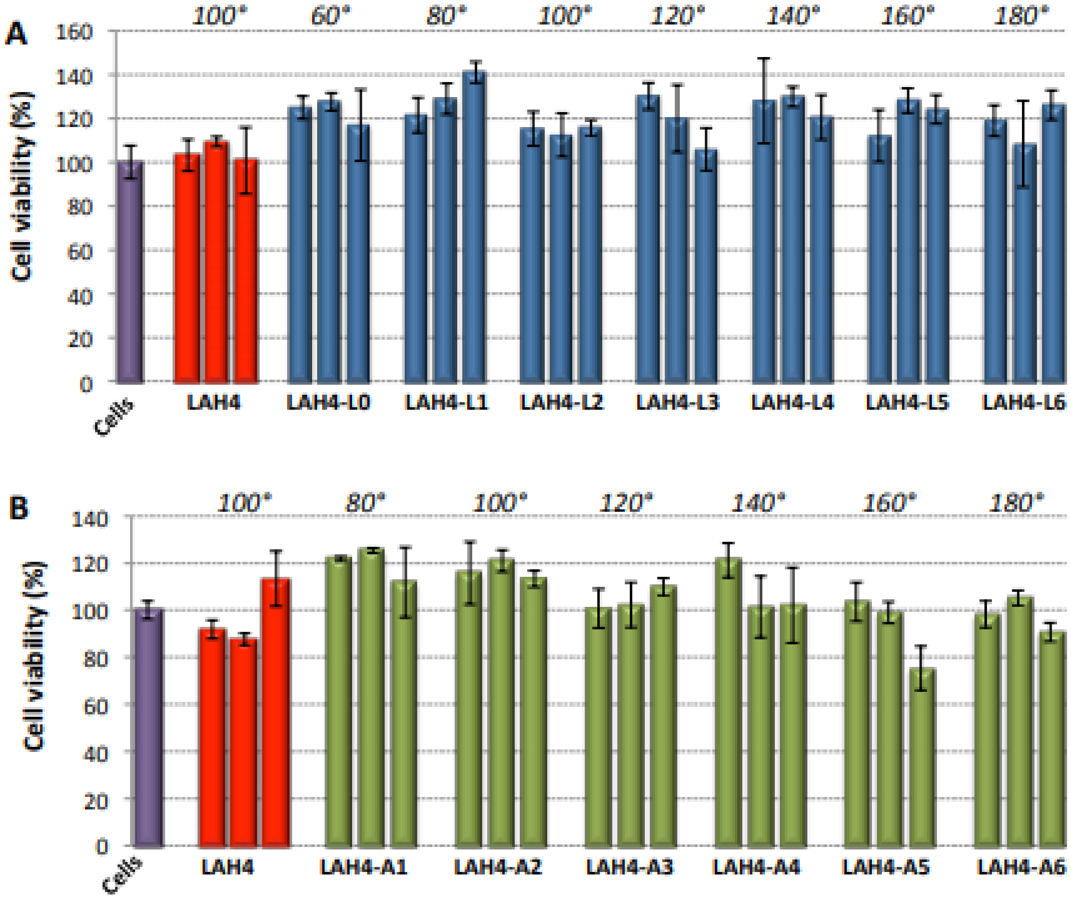

Figure S3. Peptide toxicity. HCT116 cells plates in 96-well plates were treated with LAH4 peptides (LAH4-Ln series in **A** and LAH4-An series in **B**). After 2h 30min of incubation in serum-free conditions, the medium was replaced with fresh one and the experiment was allowed to proceed. Two days later an MTS assay was performed. The cell viability efficiency is expressed as percent compared to untreated cells (=100%). Error bars represent the standard deviation of the mean. For each peptide 3 conditions were tested: 4.05, 5.4 and 8.1 µg of peptide/triplicate. Untreated cells (=100%; purple bar) were used as control. In italics are shown the hydrophilic angles.

Table S1. MIC_{50} values for the LAH4-Ln and LAH4-An peptides from fits of the data shown in Figure 3 using Origin and the function $y = y_{min} + \frac{y_{max}-y_{min}}{1+\left(\frac{x}{x_{50}}\right)^{p}}$.

LAH4-Ln Peptides	MIC_{50}	LAH4-An Peptides	MIC_{50}
-L0	3.1 ± 0.3		
-L1	8.6 ± 1.6	-A1	13.9 ± 1.6
-L2	4.7 ± 0.9	-A2	5.0 ± 1.9
-L3	1.4 ± 0.5	-A3	7.5 ± 1.5
-L4	0.8 *	-A4	6 *
-L5	1.2 *	-A5	5.3 ± 0.5
-L6	0.9 ± 0.15	-A6	2.9 ± 0.5

* the error is estimated to be in the range of the 1.5-fold dilution steps.
